# Transcultural Comparison of Mental Health and Work–Life Integration Blurring in the Brazilian and Spanish Populations during COVID-19

**DOI:** 10.3390/jpm13060955

**Published:** 2023-06-05

**Authors:** Juanita Hincapié Pinzón, Andressa Melina Becker da Silva, Wagner de Lara Machado, Carmen Moret-Tatay, Manoela Ziebell de Oliveira

**Affiliations:** 1 Escuela de Doctorado, Universidad Católica de Valencia San Vicente Mártir, San Agustín 3, Esc. A, Entresuelo 1, 46002 Valencia, Spain; 2 Postgraduate Psychology Program, School of Health Sciences, Pontifical Catholic University of Rio Grande do Sul, Porto Alegre 90619-900, RS, Brazil; 3 Faculty of Psychology, University of Sorocaba, Sorocaba 18023-000, SP, Brazil; 4 MEB Laboratory, Faculty of Psychology, Universidad Católica de Valencia San Vicente Mártir, 46100 Valencia, Spain

**Keywords:** mental health, role blurring, technology, pandemic, work, COVID-19

## Abstract

The study aimed to compare the impact of Role Blurring on mental health and Work-Life Integration in the Brazilian and Spanish populations during COVID-19. Role Blurring, which is related to resources and demands in the work context, affects coping with stressors arising from role overlapping and impacts individuals’ perception of work overload and mental health. The sample consisted of 877 adults from Spain (*n* = 498) and Brazil (*n* = 372), and various statistical analyses were conducted to compare the groups. Results showed that Role Blurring is linked to symptoms of anxiety, depression, and stress, as well as suicidal ideation. Therefore, it is essential to promote working conditions that limit expectations on availability and favor disconnection from work during leisure time. Public policies that intervene, promote, and prevent psychosocial risk factors in emergent contexts are crucial to prevent suicidal ideation and attempts. Considering the high expected influence of Blurring as a focus of interventions can be reflected in the medium term in the indicators of well-being and satisfaction of companies, institutions and organizations. This can result in the reduction of health costs aimed at cushioning post-COVID-19 impacts on mental health. The study is relevant to understand the impact of the pandemic and technology on mental health and suggests the need for interventions to promote work-life balance and prevent psychosocial risks.

## 1. Introduction

The COVID-19 pandemic has led to significant changes in the lifestyle habits of people around the world [1,2,3]. To curb the spread of the disease, measures such as physical distancing, hand hygiene, mask-wearing, and in some cases, lockdowns were implemented [2,4]. Each country developed its own criteria for managing the pandemic based on the evolving situation and the policies of their respective governments and institutions.

In Brazil and Spain, both countries experienced high rates of COVID-19 infections and deaths, and their responses to the pandemic were slow to materialize. The pandemic placed significant pressure on the Spanish healthcare system because of two key factors: the elevated vulnerability of the elderly population, and the country’s emphasis on sociability and socializing [5,6]. As of 24 January 2023, Spain had recorded the 12th highest number of confirmed COVID-19 cases globally, with 13,722,677 cases and 118,183 deaths. As of 24 January 2023, Brazil is the fifth country in the world with the highest number of COVID-19 infections, with 36,677,844 people testing positive and 695,615 deaths. These data help to explain why Brazil was chosen for the present research. After the COVID-19 outbreak, both Brazil and Spain implemented vaccination campaigns to combat the virus and protect their populations. Although facing different challenges, both countries are committed to achieving widespread vaccination coverage to control the spread of COVID-19 and mitigate its impact on public health.

In this scenario, the pandemic led to an increase in remote or telework, contributing to an increased use of technological devices [7,8]. Although this approach can be positive in maintaining employment during a public calamity, it can also have negative effects, such as overexposure to social media [9], increased fear of COVID-19 [10], increased work demands, anxiety, stress, depression, among others [11]. The financial crisis resulting from job loss and social distancing during the COVID-19 quarantine was a risk factor associated with suicidal attempts and suicide, impacting mental health [12]. It should be noted that the accumulation of work resulting from an increase in demands is promoted, accepted, and incentivized by technological connectivity that contributes to the diffusion of work into the home, turning the digital environment into an extension of labor [13]. When roles, work, and personal life overlap and there is no clear boundary between them, the so-called Role Blurring occurs [14]. This can be stimulated within the work context, in the movement of always seeking better performance. It can also be implicit and reinforced by the professional and/or coworkers themselves [14,15]. Implementing boundaries, despite not being a mediator, systematically minimizes conflict between roles [7].

The workforce in contemporary societies has undergone substantial changes, with a growing prevalence of role blurring observed in diverse domains. Any occupation that can incorporate virtual aspects is susceptible to experiencing this blurring. As a result, numerous professions across industries are facing significant challenges. This convergence necessitates individuals to possess a wider range of skills, as they must adapt to emerging technologies and remain well-informed about constantly evolving industry trends. Ultimately, the blending of job roles underscores the importance of ongoing learning and adaptability in the midst of a progressively dynamic and interconnected work environment, particularly after the COVID-19 outbreak [15]. Role Blurring is related to mental health, as role overlap can cause an overload of tasks and increase levels of stress, anxiety, depressive disorders, and other factors indicating harm to subjective well-being [16,17]. Remote work’s association with job demands is worth mentioning. Research indicates that excessive work demands can have negative repercussions on physicians, such as strained relationships, and even suicidal thoughts, among others [18,19].

Nevertheless, in the study by Ortiz-Bonnin et al. [20] in Spain, with 167 participants at three different points during the pandemic, there was no significant relationship between subjective well-being and work performance. Employees’ satisfaction with companies’ response to help them generate a balance between work and life during COVID-19 was associated with well-being and improvement in performance. The study shows that good organization favors the balance between work and personal life, which was previously considered a luxury and has been highlighted as a necessity during the pandemic. In this particular setting, leaders who maintain closer proximity to their employees exhibit a heightened ability to discern their individual characteristics and assist them in effectively navigating the demarcation line between professional obligations and personal life [21]. Chu et al. [22] conducted a study with 500 participants during the pandemic context and identified three possible protective factors to decrease stress: 1. Company support for working from home; 2. Superior’s trust in the employee; 3. Work-life balance. The impact of these on employees’ psychological well-being was considered, with only the third being statistically significant. Furthermore, interestingly, engaging in non-work-related activities while working for hours does not interfere with productivity; on the contrary, it increases it.

Thus, understanding the extent to which the pandemic context brought significant changes, Brazil and Spain were chosen as countries for data collection due to their significant differences in contexts and high number of deaths. Considering the delay in adopting effective measures to control the pandemic in one and the unknown virus and high infection rates for various reasons in both, and considering that Role Blurring can affect people’s mental health, this study aimed to answer the following question: Did Role Blurring interfere with aspects of mental health in different countries during the COVID-19 pandemic? Therefore, the objective was to expand knowledge about Role Blurring and its relationship with work overload, social support, work context, fear of COVID-19, symptoms of anxiety, depression, stress, and suicide risk.

## 2. Methods

The present study is a cross-sectional design with a quantitative approach. Data were collected through an online survey from September to December of the year 2021, during which some countries, after substantially decreasing restrictions, resumed measures to contain the sixth wave of COVID-19 due to the Omicron variant. Frequency and proportion analyses, as well as *t*-tests, were conducted to compare the means between Brazil and Spain. Then, statistical proportion tests and ANCOVA with Post Hoc Games-Howell test at a 95% confidence level were performed to compare means between three groups (suicide attempt before and after the pandemic and people without suicide attempts in each country) without assuming homogeneity of variances and using the country variable (Brazil—Spain) as a covariate. Finally, network analyses were conducted to describe the relationship between workload overload, work context, Blurring, perceived social support, fear of COVID-19, and symptoms of anxiety, depression, and stress. 

### 2.1. Participants

The current study used data from two samples of adults residing in Spain and Brazil, aged 18–70 years with a mean age of 24.88 ± 9.68. The sample size was 877 adults, with 56.7% (*n* = 498) from Spain and 43.2% (*n* = 372) from Brazil. The Spanish participants’ age ranged from 18 to 61 years (*n* = 113; 23.7% men, *n* = 363; 76.2% women; mean = 24.8 years, SD ± 9.68). The Brazilian participants’ age ranged from 18 to 61 years (*n* = 89, 24.7% men, *n* = 271; 75.2% women; mean = 34.5 years; SD ± 9.51). The descriptive analysis revealed that 75.2% (*n* = 634) of the sample were born as females and 23.0% (*n* = 202) as males. Most Spanish participants, 71.9% (*n* = 358), resided in the Valencian Community, whereas 38.5% (*n* = 146) of Brazilian participants lived in Rio Grande do Sul. The data was collected in 2021, during which 21.8% (*n* = 78) of Spanish participants and 67.5% (*n* = 175) of Brazilian participants were working from home. Participants voluntarily agreed to participate in the study without any financial compensation. Participants were required provide a nickname, ensuring their anonymity. The data was carefully examined to prevent any duplicate entries. Finally, participants received feedback on their results upon completion.

### 2.2. Instruments

A sociodemographic and labor questionnaire was used, which included information on gender, age, suicide attempt, among others, related to the use of technologies for work and their impacts on the participants’ lives. Additionally, the Spanish or Portuguese version of the Role Blurring scale, DASS-21, Work Overload, Work Context, Social Support, and Fear of COVID-19 were used.

#### 2.2.1. Fear of COVID-19 Scale (FCV-19S)

The Fear of COVID-19 Scale (FCV-19S) adapted by Martínez-Lorca et al. [23] was used. This scale was created by Ahorsu et al. [24] and measures the severity of individuals’ fear of COVID-19. It has seven items and a stable one-dimensional structure with robust psychometric properties. The factorial loads (0.66 to 0.74) and the corrected item-total correlation (0.47 to 0.56) of the COVID-19 Fear Scale were acceptable in the Spanish population. In Brazil, the validation of the scale to measure the fear of COVID-19 (FCV-19S), by Silva et al. [25], presented a standardized Cronbach’s alpha (α = 0.91) and the McDonald Omega (ω = 0.90). All items had loads higher than 0.70.

#### 2.2.2. Depression Anxiety Stress Scales (DASS-21)

The DASS-21 has 21 items, and this instrument has presented adequate psychometric properties in previous validation studies [26,27,28] and an acceptable fit to a three-factor model in Spanish-speaking samples [29,30,31]. Its internal consistency ranges from 0.70 to 0.89. The instrument was adapted to Brazilian culture by Vignola and Tucci [32]. Each subscale is composed of seven items. The depression subscale has a precision index (α = 0.92) for Brazil and (α = 0.84) for Spain; anxiety (precision index (α = 0.86) for Brazil and (α = 0.70) for Spain); and stress precision index (α = 0.90) for Brazil and (α = 0.82) for Spain.

#### 2.2.3. Scale of Work Context Evaluation (SWCE)

The scale was constructed and validated by [33] to evaluate difficulties in the work context. The scale consists of 30 items and was validated in this study for the Spanish population. The scale is divided into three factors: Work organization (α = 0.89) for Brazil and (α = 0.84) for Spain, covering the division of labor, formal rules, time, rhythms, controls, and task characteristics. Work context or work conditions (α = 0.72) for Brazil and (α = 0.88) for Spain, covering the physical environment, instruments, equipment, raw materials, and organizational support. Socio-professional relationships (α = 0.87) for Brazil and (α = 0.90) for Spain, covering hierarchical interactions, collective intra- and inter-group interactions, and external interaction. 

#### 2.2.4. Work Overload Scale

The Spector and Jex scale [34], adapted for the Brazilian context by Gabardo-Martins [35]. Like the scale for measuring Work Context, this scale was validated for Spain in the present study. It is a one-dimensional instrument consisting of five elements. Its precision index is α = 0.82 for Brazil and 0.87 for Spain.

#### 2.2.5. Work–Life Integration Blurring Scale

The scale for measuring Work-Life Integration Blurring Scale [15] is a one-dimensional instrument composed of 19 items (e.g., “Do I find it difficult to fully enjoy my leisure time due to work-related concerns?”) with a Likert response format from one to four, with (1) being the lowest frequency and (4) being the highest. The precision indices for the countries of Brazil and Spain for the Work-Family Role Blurring Scale were α = 0.94 and ω = 0.94.

#### 2.2.6. Perceived Social Support Scale (MSPSS)

The Multidimensional Scale of Perceived Social Support (MSPSS), developed by Zimet et al. [36], consists of 12 items. The Brazilian cross-cultural adaptation and validation, developed by Sousa [37], has a precision index of α = 0.93. The Spanish sample version validated by Ruiz et al. [38] has a precision index of α = 0.82 for friends, α = 0.91 for relatives, and α = 0.94 for significant others. The precision indices for Brazil were α = 0.91, α = 0.93, and α = 0.91 respectively.

### 2.3. Data Analysis

Descriptive statistical analyses were conducted, including mean and standard deviation (SD), on the variables corresponding to the instruments for the total sample and for each country, Brazil, and Spain. Sociodemographic and mental health factors, as well as the use of technology in the workplace, were examined using descriptive statistics. Differences between groups were examined using χ^2^ tests and *t*-tests for independent samples. Similarly, differences in suicide attempts before and after the onset of the pandemic were explored with the group that did not attempt suicide, adjusted for size using a one-way analysis of covariance (ANCOVA), with country (Brazil-Spain) as a covariate (independent). 

The V of Cramer and d of Cohen were used to estimate effect size. An effect size is considered small when 0.10 < V < 0.30 or 0.20 < d < 0.50, medium when 0.30 < V < 0.50 or 0.50 < d < 0.80, and large when V > 0.50 or d > 0.80 [39]. Subsequently, a network analysis was conducted. The network approach exposes the elements that interact and reinforce each other, a system of causal relationships [40]. Networks consist of nodes and lines. The nodes represent variables, and the lines connect the nodes and represent the relationship between them [41].

For the flowchart analysis, the partial correlation matrix was estimated by inverting the correlation matrix [42]. Then, the Least Absolute Shrinkage and Selection Operator (LASSO) regularization was applied to set small correlations to zero [43], using the Extended Bayesian Information Criterion (EBIC) as the model selection index, which is used to select the penalized model with the smallest residual. The differential role of variables within each network was investigated by calculating mediation and closeness centrality measures. That is, the repetitions of a node as the shortest path between two other nodes and the average distance of a node from the others. The analyses were conducted using the RStudio software (RStudio, 2012). The packages used were psych [44], qgraph v. 1.6.5 [45], igraph v. 1.2.5 [46], bootnet v. 1.3 [47], and NetworkComparisonTest [48].

## 3. Results

The results show the description of the sociodemographic data of Brazil and Spain; the analyses related to *t*-test, Chi2, ANCOVA. Then, the correlation matrix between variables is presented, followed by the network analysis corresponding to the graphical representation of the correlation matrix, and the Flow or Flux diagram, which takes Blurring as the main variable to show the first- and second-degree relationships with the remaining variables. Finally, the centrality measures of the variables are presented. The descriptive analysis of the sociodemographic questionnaire for each country showed that of the participants, 93.0% (*n* = 306) of Spanish and 90.1% (*n* = 210) of Brazilians were vaccinated against COVID-19. Regarding their employment situation, 36.8% (*n* = 176) of the Spanish participants and 85.5% (*n* = 196) of Brazilians were in the labor market at the time of data collection; 21.8% (*n* = 78) of Spanish individuals and 67.5% (*n* = 175) of Brazilians ones were working from home or in a different work environment than usual at the time of collection; and 74.1% (*n* = 126) of Spanish and 64.7% (*n* = 125) of Brazilians were satisfied with their job.

Regarding mental health, 54.6% (*n* = 94) of Spanish and 95.6% (*n* = 88) of Brazilians felt that the level of productivity demanded negatively affected their mental health; 67.5% (*n* = 269) of Spanish and 57.9% (*n* = 156) of Brazilians felt that technological devices (mobile phone and its applications, laptop, email) as a means of performing work generated disadvantages in their lives, and 84.1% (*n* = 143) of the Spanish respondents and 68.4% (*n* = 63) of the Brazilians ones reserved leisure time in their study or work routine. Confirming the Kolmogorov-Smirnov or Shapiro-Wilk test, we can determine that the samples do not meet the normality condition because the *p*-value < 0.005 except for the variable Work Context. Next, we verified the results of the Levene’s test to examine the equality of variance. The Levene’s test *p*-value <0.005 for the Role Blurring variable suggests that the equality of variance condition is also not met. Thus, the results of the independent *t*-tests for the description of the scale scores and the difference in means between the countries (Brazil and Spain) are illustrated in Table 1. 

The analysis indicates that the Spanish participants showed higher levels with a small effect size in the perception of Social Support. In turn, the Brazilian participants showed higher levels of fear of COVID-19, greater perception of diffuse boundaries between life and work, more symptoms of anxiety, depression, and stress, greater difficulties in the work context, and more work overload during the time of data collection.

The Table 2 shows the differences in proportion of mental health variables by sample. The χ^2^ analysis showed that during the data collection period, more Brazilians were receiving psychological care and had more psychiatric treatment than expected compared to the Spaniards, with a small effect size, indicating that it is not necessarily a trend. Similarly, proportionally more Brazilians have a family history of mental disorders and also had more suicidal ideation, although this last association is not as strong. Despite this, there is a difference of more than 95% in the prevalence of suicidal ideation compared to Spaniards. However, despite having more suicidal ideation, Spaniards were the ones who attempted suicide more frequently.

Regarding suicide attempts before or after the start of the pandemic, the majority (Brazilians and Spaniards) were before the start of the pandemic, with a significant difference and a not very large effect size. Similarly, for both Brazilians and Spaniards, the majority of suicide attempts were before the start of the pandemic. However, proportionally, the number of suicide attempts after the start of the pandemic was higher for Spaniards.

It is worth highlighting that a prevalence difference test is not being conducted, what is being identified is how much the prevalence is related to the country variable and that this nominal difference is not only explained by the country of origin. This will be evidenced in greater depth in the network analyses.

Considering that 9.85% of the total sample attempted suicide at some point, of which 0.26% of Brazilians and 2.20% of the Spanish attempted it after the start of the pandemic, the following analysis will identify the factors that separate the groups, regulating the effect of the country variable. Due to the small number of cases, the entire sample was used so that differences in responses due to the respondents’ own characteristics could be considered. That is, with few cases there is a distortion in the measure of variability. Given the small number in the suicide attempt group, no comparisons were made. The groups are examined in relation to the variables of interest considering the absence (without suicide attempt) or presence of suicide attempts before and after the start of the pandemic. The results are presented, in sequence, in Table 3. 

The difference between groups was significant in the variable related to fear of COVID-19 for the group without suicide attempt and those who attempted suicide after the start of the pandemic. There were also differences between the group without suicide attempt and those who attempted before the start of the pandemic in symptoms of anxiety, depression, and stress. 

### Network Analysis

The figures below represent graphical objects (graphs) corresponding to the matrix of regularized partial correlations through which the associations between variables (nodes) are shown. The relationship between the nodes is shown with edges (lines) in blue for directly proportional associations and red for inversely proportional ones. The thickness of each line determines the magnitude of the association between the nodes.

**Figure 1:**
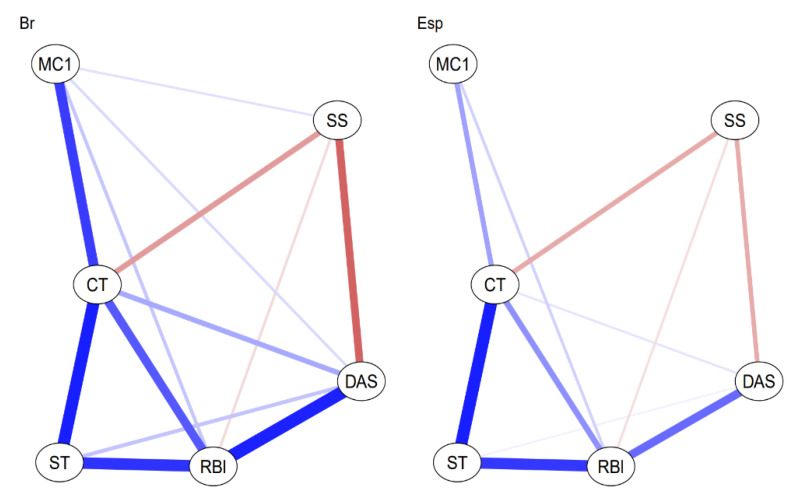
Analysis of Invariance in the Network Composed of Scales. Note: The network consists of six nodes, with 13 and 11 edges respectively, representing conditional dependencies. This is a Gaussian graphical model (GGM) because the nodes are multivariate normal variables. Abbreviations: MC1 = Fear of COVID-19, CT = Work Context, ST = Work Overload, RBl = Role Blurring, DAS = Scale for measuring anxiety, depression and stress DASS-21, SS = Social Support.

Figure 1 shows the networks with the scales in each country. The invariance in the structure of both networks is observed comparatively, which will allow for a better understanding of the variations in the network composed of the scales and sociodemographic variables, regarding the sensation of loss of control of time during confinement and suicidal ideation. This ensures clarity in the nature of the differences and similarities between contexts and rules out possible bias in the application of the scales.

The centrality measures in both countries aim to investigate the network at the level of global strength (the same overall level of connectivity) and individual nodes. That is, each specific node is identical in all cohorts. Node strength invariance was only tested because the lack of network structure invariance was confirmed. For all comparisons of variable centrality measures, *p* > 0.05 was observed. Table 4 presents the centrality measures of the variables. 

**Figure 2:**
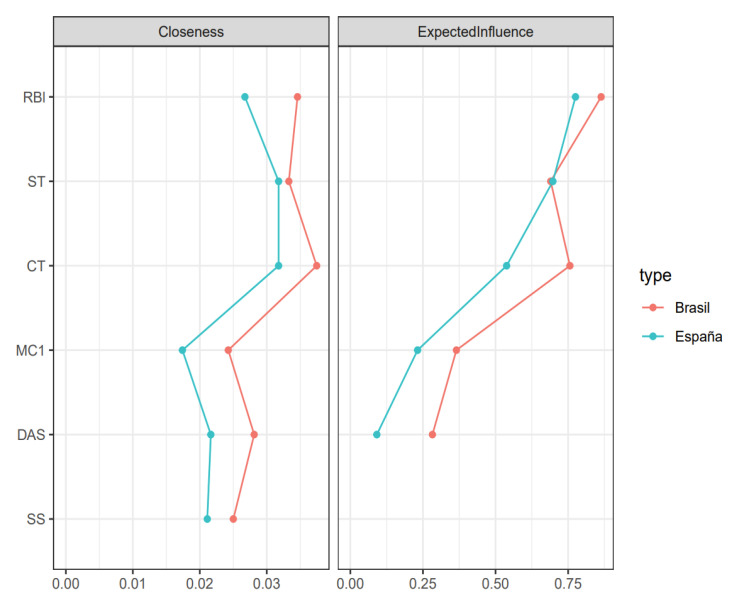
Standardized Analysis of Centrality Measures and Expected Influence. Note: The red line represents the network estimates for the scales from Brazil; the green line represents the network estimates for the scales from Spain. Abbreviations: MC1 = Fear of COVID-19, CT = Work Context, ST = Work Overload, RBl = Role Blurring, DAS = Scale for measuring anxiety, depression and stress DASS-21, SS = Social Support.

The comparative analysis of centrality measures between nodes (Closeness) and expected influence of the scales is presented in Figure 2. The variable that was most correlated with the others, for Brazil and for Spain, in the centrality measures of the scale invariance network was the work context (CT), and the one that most influenced the network as a system was Role Blurring (RBl) in both countries.

**Figure 3:**
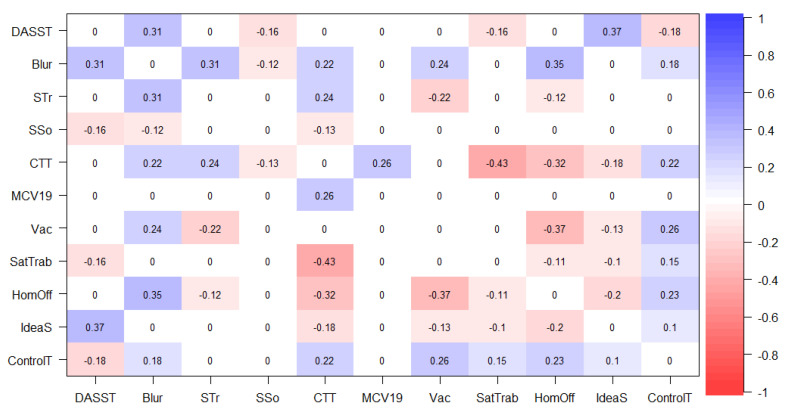
GeLasso Method, Partial Correlation Matrix of Scales and Sociodemographic Variables in Brazil. Note: SSo = Social Support, MCV19 = Fear of COVID-19, STr = Work Overload, DASST = Anxiety, Depression and Stress Symptoms, Blur = Role Blurring, CTT = Difficulties in Work Context, HomOff = Working in Home Office mode, SatTrab = Job Satisfaction, ControlT = Sense of Loss of Control of Time during Confinement, Vac = Being vaccinated, IdeaS = Suicidal ideation.

**Figure 4:**
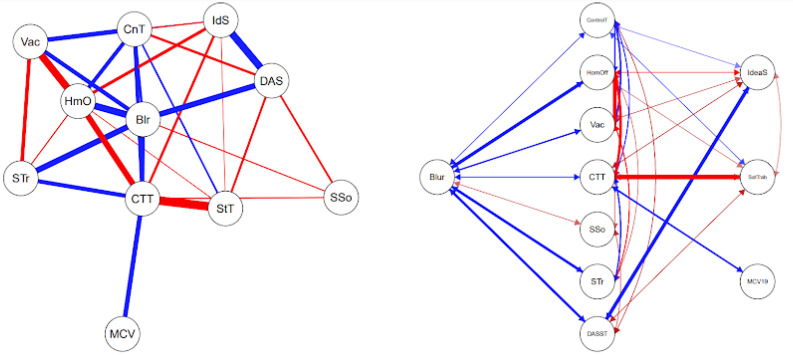
GeLasso Method, Network Analysis and Flowchart. Correlation Scales and Sociode-mographic Variables in Brazil. Variable: Role Blurring. Note. SSo = Social Support, MCV = Fear of COVID-19, STr = Work Overload, DAS = Symptoms of Anxiety, Depression and Stress, Blur = Role Blurring, CTT = Difficulties in the Work Context, HmO = Working in Home Office Mode, StT = Job Satisfaction, CnT= Feeling of Loss of Control of Time during Confinement, Vac = Being Vaccinated, IdS = Suicidal Ideation, CnT = Feeling of Loss of Control of Time during Confinement, Vac = Being Vaccinated, IdS = Suicidal Ideation.

**Figure 5:**
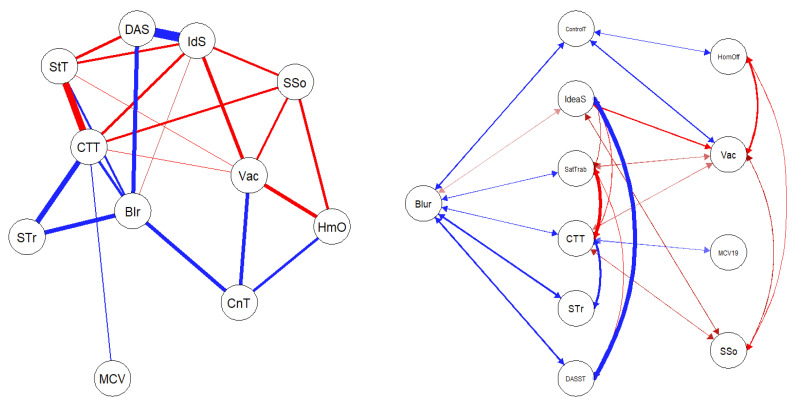
GeLasso Method, Network Analysis and Flowchart. Correlation of Scales and Sociodemographic Variables in Spain. Note: SSo = Social Support, MCV19 = Fear of COVID-19, STr = Work Overload, DASST = Anxiety, Depression and Stress Symptoms, Blur = Role Blurring, CTT = Difficulties in Work Context, HmO = Working in Home Office mode, SatTrab = Job Satisfaction, ControlT = Sense of Loss of Control of Time during Confinement, Vac = Being vaccinated, IdeaS = Suicidal ideation.

The following figures represent graphical objects (graphs) corresponding to the matrix of partially regularized correlations that show the associations between variables (nodes). The relationship between the nodes is shown with edges (lines) in blue for directly proportional associations and in red for inversely proportional ones. The thickness of each line determines the magnitude of the association between the nodes. Figure 3 and Figure 4 represent the partial correlation matrices of each country (Brazil and Spain) corresponding to the associations between scales, sociodemographic data, and suicidal ideation for each one. Secondly, Figure 4 and Figure 5 show the graphs derived from the correlation matrix. The correlational nature of the associations in this analysis is emphasized, which in no way implies a causal relationship. In Figure 3, which represents the partial correlation matrix of the networks, it is possible to perceive that for the Brazilian context, there was a positive relationship between Role Blurring (Blr), working from home (HmO), symptoms of anxiety, depression, and stress (DAS), work overload (STr), difficulty in the work context (CTT), and the sensation of loss of control of time during confinement (CnT). While in terms of strong negative relationships, the relationship between difficulty in the work context (CTT) and job satisfaction (StT), and between being vaccinated (Vac) and working from home (HmO) is evident (highlighting that this later has a positive relationship with Role Blurring). In this way, it is understood that indirectly, the increase in vaccination may have resulted in more people in the Brazilian population returning to in-person work, and therefore reduce Role Blurring and possibly improve mental health indicators.

**Figure 6:**
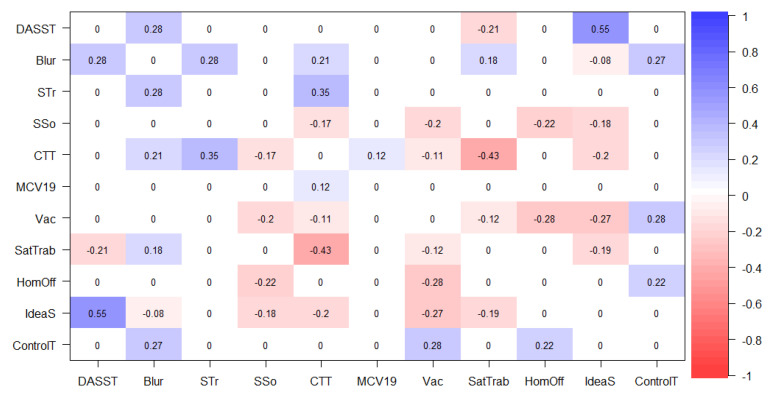
GeLasso Method, Network Analysis, and Flowchart. Correlation of Scales and Sociodemographic Variables in Brazil. Note: SSo = Social Support, MCV = Fear of COVID-19, STr = Work Overload, DAS = Anxiety, Depression and Stress Symptoms, Blur = Role Blurring, CTT = Difficulties in Work Context, HmO = Working in Home Office mode, StT = Job Satisfaction, CnT = Sense of Loss of Control of Time during Confinement, Vac = Being vaccinated, IdS = Suicidal ideation.

Th Flowchart or diagram of the Brazilian sample (left side) in Figure 6 is interesting to observe. It shows that job satisfaction (SatTrab) is indirectly related to Role Blurring (Blr) when mediated by the positive relationship between Blr and difficulties in the work context (CTT). Fear of COVID-19 (MCV19) is also positively related to Blr when positively mediated by CTT. Suicidal ideation (IdeaS) in second-order variables (mediated by others) is related to Blr from the direct positive relationship of Blr with DAS. That is, IdeaS, SatTrab, and MCV19 form a second-degree line or indirect relationships with Blr, while the other variables are given by direct association estimates.

In Figure 6, which represents the data of partial correlation between the scales and sociodemographic variables in Spain, a strong positive relationship is observed between symptoms of anxiety, stress, and depression (DAS) and suicidal ideation (IdS), as well as a strong negative relationship between job satisfaction (StT) and difficulties in the work context (CTT). A moderate, but noteworthy relationship is that being vaccinated (Vac) is negatively associated with IdS. Similarly, the subset of positive relationships between difficulties in the work context, Role Blurring (Blr), and work overload (STr) stands out. On the other hand, as expected under the health safety conditions offered in the Spanish territory, being Vaccinated was negatively correlated and working from home (HmO) was positively correlated with the sense of loss of control of time during confinement (CnT).

The network (right-hand side) and flowchart (left-hand side) graphs of the Spanish sample in Figure 5 show that the variable HmO is associated with Blr when positively mediated by the feeling of loss of control of time during confinement. Fear of COVID-19 is associated with Blr mediated by the positive association of CTT. On the other hand, there is a negative relationship between Vac and Blr when mediated by IdeaS. Finally, it is interesting to note that Blr is positively associated with CTT, and the relationship between CTT and perceived social support is inverse. The relationship between Blr and HmO, Vac, MCV19, and SSo is of second order, while with other variables, it is direct. It is noteworthy that the association between DAS and IdeaS is inverse, i.e., Blr and IdeaS have a negative relationship, and the relationship between Blr and DAS is positive.

## 4. Discussion

Starting with a comprehensive examination of the overall landscape using descriptive data and network analysis, the discussion of the results will progress towards their correlation with mental health specificities derived from traditional analyses of variance. Consequently, the findings of this study, obtained during the sixth wave of the COVID-19 pandemic, reveal that most Brazilian and Spanish respondents expressed satisfaction with their work during the survey period. Among the respondents, 32.4% of Spanish individuals and 21.8% of Brazilians ones were engaged in remote work. However, when respondents were asked more detailed questions, Brazilians reported encountering more difficulties in the work context, experiencing greater role blurring, and facing higher work overload.

Delanoeije et al. [49] conducted a study utilizing the Boundary Theory framework, where they found evidence supporting the notion that teleworking days were associated with increased conflict between work and home domains. Specifically, their analysis revealed a positive relationship between network nodes representing Role Blurring (Blr) and difficulties in the work context (CTT), indicating that teleworkers experienced challenges in managing the boundaries between their professional and personal roles. Moreover, the researchers observed that the positive associations between network nodes of work overload (STr) and symptoms of anxiety, depression, and stress (DAS), as well as between CTT and DAS, were mediated by Role Blurring (Blr) within the network.

The rise of virtual or hybrid work environments, which has been further accelerated by factors such as the COVID-19 pandemic, has resulted in negative consequences, including phenomena like video conferencing fatigue, akin to the broader concept of work fatigue [50]. Existing literature has also documented detrimental effects associated with telework, such as overload stemming from interruptions caused by the use of Information and Communication Technologies (ICT), work-family conflict, and techno-stress [51]. In essence, teleworking days impact employees’ work-home conflict through mediating mechanisms involving transitions between work and home domains, as highlighted by Delanoeije et al. [49].

Emerging forms of work demands resulting from digitization, such as techno-stress and telepressure, have been found to have detrimental effects on job satisfaction and commitment, ultimately impacting employee well-being [52]. This is evident in the networks of two countries, where a positive correlation between job satisfaction (SrT) and the feeling of loss of control of time during confinement (CnT) [53] suggests that while working from home (HmO) offers greater flexibility and control over work location and timing, it also intensifies network communication through Information and Communication Technologies (ICT), raising expectations of availability and reducing the ability to disconnect from work. The association between variables is supported by the scales of Work-Life Integration Blurring [15], Work Overload [34,35], and Work Context [33], which demonstrate interconnected items and underline the importance of this relationship. Central measures indicate that the feeling of loss of control of time during confinement (CTT) exhibits the highest correlation with the other variables, while the variable Blr is identified as a potential focal point for intervention.

Among the sociodemographic variables, remote work (home office) emerges as significant. Jaiswal et al. (2022) suggest that adapting to the shift from in-person work to virtual work can initially induce stress but later foster resilience and cognitive adaptations. Vaccination also demonstrates interesting associations, highlighting the importance of public policies that promote and prioritize vaccination efforts. 

According to Royo [54] and Henriques and Vasconcelos [55], for different reasons, the governments of Brazil and Spain might not have adopted effective measures regarding the COVID-19 pandemic. Brazil, despite having a historically effective Unified Health System regarding vaccination campaigns, had the dissemination of contradictory information. The federal and state governments disagreed on the implementation of measures, generating a lack of unity in the territory and with protocols of the World Health Organization (WHO) [56]. This lack of unified guidelines, the spread of fake news, among other things, generated disbelief, increased population fear of vaccines, and directly affected the number of COVID-19 infections, the high number of deaths, as well as the implications for socioeconomic, mental health markers and prolonged confinement time.

Furthermore, the results showed that Brazilians had more fear of COVID-19, which may be related to the fact that at the time of data collection, denialism, and resistance of some local governments in Brazil to follow scientific-based health recommendations were still claiming lives [56]. On the other hand, during the same period, Spain had already lifted restrictions and had a significant advantage in proportion to the control of deaths, as demonstrated by the WHO figures. All these circumstances may have influenced the fact that Spanish individuals reported a greater perception of social support, and Brazilians, compared to them, had a higher incidence of anxiety, depression, and stress symptoms.

In terms of sociodemographic variances, it is noteworthy to acknowledge that the survey included a higher proportion of Brazilian participants engaged in formal employment, while most Spanish respondents were primarily university students. There may also be differences in cultural aspects concerning labor demands and resources, as well as the context and guidelines in emergency situations. Similarly, variations in the appearance of virus infections, with different incidence rates from continent to continent, may have influenced variables such as suicidal ideation. These variations could be related to individual characteristics, cultural factors, and pandemic management in each country [57].

The alteration of working hours through ICT (Information and Communication Technology) tends to blur boundaries outside of working hours due to constant time and location availability. This can result in pressure, physical and mental fatigue [58]. Such fatigue may be related to the feeling of losing control over working time during confinement, which is referred to as ControlT. The network Flow diagram, for example, illustrates the significance of ControlT and its direct relationship with Role Blurring (Blur).

Furthermore, this study identifies the transcultural impact of Work-Life Integration Blurring on mental health during COVID-19 as a central finding. Unlike the Brazilian sample, which did not show a direct association between Blr and Suicidal Ideation (Ids) in the last 30 days prior to responding to the survey, the Spanish sample exhibited a negative relationship between these variables. It is noteworthy that in both samples, there was a positive correlation between Blr and DAS (anxiety, depression, and stress), as well as between DAS and IdS. This demonstrates an increase in symptoms of anxiety, depression, and stress when there is an increase in role overlap and a sense of losing control over time. Additionally, in the Spanish sample, there was a direct relationship between Blr and IdeaS.

As the Role Blurring decreases, suicidal ideation increases in the Spanish sample, highlighting the possibility that Spanish respondents feel more “guarantees” of disconnection compared to Brazilians; this is due to the provisional measures promoted by the pandemic proposed in Royal Decree Law 28/2020 of September 22, which established, among other things, a time limit on work and the right to digital disconnection. This Decree was replaced by Law 10/2021 of July 9, on remote work. In this context, of awareness about mental health in the digital era labor world, the Resolution of the European Parliament of 5 July 2022, was born as a response to the need to regulate the process of digitization of work environments as a possible risk to workers’ mental health. Nevertheless, and despite the effort of the European Parliament to regulate the use of ICTs in the work environment, the COVID-19 pandemic has caused mental health effects still to be measured [59].

Role blurring does not necessarily become a problem during confinement, unlike the feeling of a loss of control over time, which is revealed as a problematic variable. This is considering the relationship between job satisfaction (StT) and being vaccinated (Vac), as well as Vac with working from home (HmO), both of which are negatively correlated. Spanish participants possibly consider working from home advantageous. In their research on working conditions in Europe, Palumbo et al. [60] found that telework, or working from home, despite improving the flexibility of work agreements, blurs the boundaries between work and daily life, generating stress in managing such interaction. However, when a mediator is present, HmO increases motivation and job satisfaction, promoting a positive perception of work-life balance.

Emphasizing the significance of sociodemographic variables in analyzing scale scores across different countries is crucial. By establishing equivalence in the structure of networks comprising only the scales, the relevance of sociodemographic variations becomes clearer in the comprehensive network of Figure 3 and Figure 4, which includes both scales and sociodemographic variables. Specifically, variables such as vaccination status (Vac), remote work (HmO), and suicidal ideation (IdeaS) play a significant role in each sample, shedding light on the impact of these sociodemographic data on mental health. To gain a better understanding of the effects of the analyzed factors on mental health and to assess the stability of the presented model, specific differences were explored. These differences included psychological support, psychiatric treatment, a history of mental disorders in the family, and suicidal ideation. Interestingly, it was observed that Brazilians exhibit higher proportions in all of these factors compared to Spanish group. Notably, research conducted by Dhrisya et al. [61] warns about the influence of social and biological parameters on suicidal ideation during the pandemic. Their study revealed that psychosocial factors, such as unemployment, lack of support, fear, stress, and anxiety, exacerbated suicidal ideation in the context of a pandemic. These factors, in conjunction with behavioral and neurobiological elements, contribute to suicide attempts.

The graphical representation of the network in both samples highlights the strong correlation between suicidal ideation (IdeaS) and symptoms of anxiety, depression, and stress (DAS). This correlation is directly associated with the variable “Blr” and reveals a significant difference of over 95% in the prevalence of suicidal ideation between Brazilians and Spanish respondents. Despite Brazilians having a higher proportion of suicidal ideation, Spanish participants had a higher rate of suicide attempts. It is worth noting that most suicide attempts by both Brazilians and Spanish individuals occurred before the start of the pandemic. However, when considering the proportion, Spanish participants exhibited a higher rate of suicide attempts after the onset of the pandemic, surpassing expectations. The nominal difference in prevalence is likely attributed to other variables, not necessarily the country of origin. Nevertheless, it is evident that the prevalence of the analyzed mental health variables is related to the country variable. According to Rothman and Sher [57], the consequences of the pandemic on suicide rates vary based on factors such as public health control efforts, patient demographics, telehealth availability, and existing supportive infrastructure within each country. Considering this premise, it becomes apparent how the first wave of the pandemic caught the Spanish population and healthcare system off guard. Therefore, it is crucial to gather more data in each context to conduct longitudinal research designs that can verify potential trends over time, particularly after a significant return to in-person work.

It should be noted that self-administered questionnaires, which are commonly utilized for research and data collection, have inherent limitations that need to be acknowledged. One of the main drawbacks is the possibility of response bias, wherein participants may provide inaccurate or incomplete answers due to factors such as social desirability bias or misunderstanding the questions. Moreover, the absence of a trained interviewer means that there is no opportunity to address any uncertainties or ensure consistent comprehension of the questionnaire. Another limitation arises from the reliance on self-reporting, which can be influenced by subjective interpretations, personal biases, or the responder’s emotional state at the time of providing the answers. Lastly, the identity of the respondents cannot be always reliably verified.

To conclude, the analysis of covariance sheds light on a significant difference regarding the fear of COVID-19. This fear is lower among individuals who attempted suicide after the onset of the pandemic, compared to those who did not make any suicidal attempts. Additionally, it is lower for individuals who attempted suicide prior to the health emergency in relation to symptoms of anxiety, depression, and stress. It is crucial to consider the increase in work demands, which leads to work overload due to work-related communication outside normal working hours and the constant expectation of connectivity [13]. These factors are further amplified by the necessity to seek alternative communication channels and find solutions for reduced educational, work, and social dynamics resulting from confinement. Similar to the impact of social distancing and financial crises on mental health, which have been identified as risk factors associated with suicidal attempts and suicide [12], the blurring of boundaries and overlap of roles between various aspects of life prove to be significant points for intervention and prevention of psychological distress in both countries

## 5. Conclusions

The factors associated with the work context, such as expected productivity and duration of the workday, contribute to the decrease or increase of psychological distress. New work dynamics related to the use of technology as a work assistant, promoter of Role Blurring, can affect work organization (formal rules, breaks, shifts, and workload), thereby increasing work overload. Therefore, it is essential to identify the associated variables and generate health promotion and prevention policies that limit workers’ availability outside working hours as risk factors associated with the imbalance between different roles due to the ease and frequency of transition generate high levels of stress.

It is important to highlight that teleworking has a relevant instrumental function and can be a resource to alleviate work demands, depending on productivity expectations. However, the relationship with work organization (types of pressure, deadlines) is crucial in the perception of work overload and the balance between personal and work domains. Providing individuals with tools to meet work demands allows for balancing roles and therefore reducing Blurring, which in turn influences the reduction of stress and prevention of risk factors in mental health.

This research aimed at a better understanding of the phenomenon of Role Blurring and its effects on mental health in the digital era. Through the analysis of the obtained data and reading from the studied contexts, it provided a comparative overview of the impact during the COVID-19 pandemic in a European country (Spain) and a Latin American one (Brazil). It is suggested to compare the effects of Blurring on mental health in professionals and students for future research, as well as specific samples of workers. It is worth noting that this study’s limitation is that there are more Brazilian workers in the sample and that the Spanish’ most frequent activities correspond to university studies. It is essential to generate longitudinal studies to review the possible causal nature of the variables and delve into protective factors that increase individuals’ resources to cope with the vicissitudes that may arise from home or work.

The associations investigated in this thesis aim to enhance the understanding of Role Blurring’s manifestations in everyday life, helping to identify and address its possible connections and consequences. It also serves as a guide for the development of public policies and the implementation of protective measures aimed at the well-being of Brazilian and Spanish citizens. In the medium term, this directly reflects in the performance indicators of companies, institutions, and organizations. By reducing health costs, these measures can also prevent phenomena such as turnover, burnout, and work-family conflict. Moreover, it will be crucial to prevent potential symptoms associated with the imbalance between work and private life, such as anxiety, depression, and stress, while promoting the well-being of the working population to mitigate the impact on the mental health of digital assistants. Thus, in the long term, the implementation of primary health strategies can lead to effective interventions against the adverse effects of Role Blurring between personal and professional life.

## Figures and Tables

**Table 1:** Description of Raw Scores of Variables, and Mean Differences between Brazil and Spain.

	Frequency (%)				*T Test*				
	Brazil		Spain						
Sample	379 (43.2%)		498 (56.7%)						
Variables	M	SD	M	SD	Test	Effect Size	t (gl)		sig.
- SWCE	77.4	21.8	67.8	19.4	Student Mann-Whitney	Cohen’s d Rank	0.46 0.23	5.36 (535)	<0.001
- Work-Life Integration Blurring Scale (RB) **	49.7	14.8	41.8	11.4	Student Mann-Whitney	Cohen’s d Rank	0.60 0.31	7.52 (634)	<0.001
- MSPSS **	63.3	14.5	67.4	13.5	Student Mann-Whitney	Cohen’s d Rank	−0.29 −0.17	−3.43 (551)	<0.001
- Work Overload Scale **	17.4	5.22	15.8	5.12	Student Mann-Whitney	Cohen’s d Rank	0.31 0.18	3.80 (594)	<0.001
- FCV-19S **	17.7	6.41	12.6	5.80	Student Mann-Whitney	Cohen’s d Rank	0.84 0.46	9.79 (552)	<0.001
DASS-21 *	21.7	13.7	18.6	13.6	Student Mann-Whitney	Cohen’s d Rank	0.22 0.14	2.84 (682)	<0.005

Note. For the Student *t*-test. effect size is given by Cohen’s d. For the Mann-Whitney test. effect size is given by the rank biserial correlation. * = *p* < 0.05. ** = *p* < 0.001.

**Table 2:** Proportion of Individuals from Each Country in Relation to Mental Health in the Samples.

Variables	Brazil (n = 372)	Spain (n = 498)	χ^2^
Are you currently receiving psychological support?	Yes—n (45.5%) No—n (54.4%)	Yes—n (22.0%) No—n (77.9%)	X^2^ = 47.9 gl = (1) *p* < 0.001 Phi = 0.24
Have you ever received psychiatric treatment with medication before starting to work or study?	Yes—n (17.5%) No—n (82.4%)	Yes—n (10.7%) No—n (89.2%)	X^2^ = 7.48 gl = (1) *p* < 0.001 Phi = 0.09
Is there a history of mental disorder in your family?	Yes—n (53.8%) No—n (46.1%)	Yes—n (31.4%) No—n (68.5%)	X^2^ = 38.9 gl= (1) *p* < 0.001 Phi = 0.22
Are you currently experiencing suicidal ideation (have you thought about killing yourself)? (Consider the last 30 days)	Yes—n (12.1%) No—n (87.8%)	Yes—n (6.42%) No—n (93.5%)	X^2^ (1) = 7.61 gl = (1) *p* < 0.006 Phi = 0.99
Have you ever attempted to take your own life?	Yes—n (8.28%) No—n (91.7%)	Yes—n (10.9%) No—n (89.0%)	X^2^ (1) = 1.47 gl = (1) *p* < 0.225 Phi = 0.043
Was it before the start of the pandemic?	Before n (96.1%) After n (3.8%)	Before n (78.4%) After n (21.5%)	X^2^ (1) = 4.11 gl = (1) *p* < 0.043 Phi = 0.23

**Table 3:** ANCOVA Kruskal Wallis and Post Hoc Games-Howell for Scales and Suicidal Attempt Before and After the Onset of the Pandemic, Full Sample of Brazil and Spain.

Sample	Frequency (n)						ANCOVA				
	Without Suicide Attempt		Before the Onset of the Pandemic		After the Onset of the Pandemic		Comparison between Groups		Countries		Kruskal-Wallis
	(n = 800)		(n = 65)		(n = 12)						
	Mean	SD	Mean	SD	Mean	SD	F (gl)	sig	F (gl)	sig	sig
- SWCE	71.6	21.5	75.5	20.4	77.8	25.0	1.11 (2;489)	0.330	29.2 (1;532)	<0.001	0.471
- Work-Life Integration Blurring Scale (RB)	44.5	13.3	49.7	14.9	45.2	10.9	4.47 (2;630)	0.012	58.2 (1;631)	<0.001	0.056
- MSPSS **	66.3	13.6	60.2	16.2	56.0	20.0	6.27 (2;547)	<0.005	13.1 (1;548)	<0.001	0.021
- Work Overload Scale **	16.4	5.24	17.3	5.07	18.3	4.83	0.76 (2;590)	0.464	14.5 (1;591)	<0.001	0.476
- FCV-19S	14.9	6.49	13.5	7.18	8.87	2.85	2.69 (2;548)	0.068	92.0 (1;549)	<0.001	<0.005
- DASS-21 **	18.4	12.9	31.8	13.2	37.7	17.4	40.5 (2;678)	<0.001	12.5 (1;679)	<0.001	<0.001

Note. Due to the limited number of cases for the suicide attempt group after the pandemic, the analysis was conducted on the total sample to identify the factors that differentiate the groups. ** = p < 0.001.

**Table 4:** Measure of centrality of the variables.

Variable 1	Variable 2	*p*-Value
DASS-21	RB	0.27
DASS-21	ST	0.47
RB	ST	0.53
DASS-21	CT	0.30
RB	CT	0.58
ST	CT	0.39
DASS-21	SS	0.50
RB	SS	0.92
ST	SS	<0.99
CT	SS	0.83
DASS-21	MC19	0.49
RB	MC19	0.98
ST	MC19	<0.99
CT	MC19	0.25
SS	MC19	<−0.99

Note. Due to the proximity to unity (1.00), the *p*-value represented was in some cases represented as <0.99.

## Data Availability

On request to the first author.
